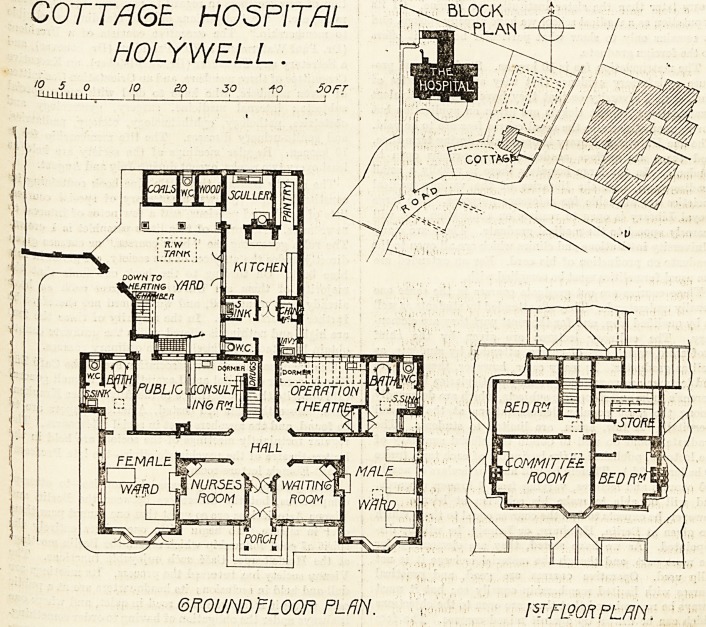# The Cottage Hospital at Holywell, Flintshire

**Published:** 1908-11-14

**Authors:** 


### HOSPITAL ADMINISTRATION.

### CONSTRUCTION AND ECONOMICS.

### THE COTTAGE HOSPITAL AT HOLYWELL, FLINTSHIRE.

We learn from the *County Herald* that for several years past the Committee of the local Dispensary have been considering the possibility of building a Cottage Hospital at Holywell. Gradually a sum of between £300 and £400 was collected; but, as a hospital would cost a great deal more than that, tne Committee did not feel justified in beginning building operations. The position of affairs reached the ears of Mr. Edwin Jones, J.P., of Clapham Park, who, being a native of Holywell and interested in the town, offered to build the hospital and present it to the town.

The main elevation faces south. It is a symmetrical building, with the entrance in the centre, the passage opening into an octagonal hall, on the right-hand of which is the waiting-room and on the left the nurses' room. A short passage running eastwards leads to the men's ward, which contains three beds, and a similar passage running westwards leads to the women's ward, which contains two beds; both wards are of the same size and shape. There is a large bay-window in the end facing south, and the side of the ward (west on the women's ward and east on the men's) has two windows. The other sides of the wards have no windows at all, consequently there will be no direct cross-ventilation; but the south windows are so large that there ought to be considerable currents of air diagonally.

Of course it is not always easy to carry out proper hospital arrangements in so small a building as is here available; nevertheless in this case it would have been decidedly better to have run the south ends of these wards several feet beyond their present line and to have continued the east and west passages through what is now the north end of the ward. At present the floor-space can hardly reach one hundred square feet per bed, and any extra space obtained would have been useful. North of the wards are the bath-rooms, closets and sinks, and unfortunately these are not cut off from the main by cross-ventilated passages. The operating-room is properly placed to the north, and has abundance of light. The kitchen and kitchen adjuncts seem conveniently arranged and compact. On the first floor are situated the committee-room, two bedrooms, and a store-room.

The wards are to be lined with green enamelled bricks; and it is stated that the operating-room will be fitted with the latest surgical appliances and that it will be thoroughly ventilated and warmed. The ward floors will be laid down with maple-wood blocks. The wards will be warmed by open fireplaces and by hot-water pipes.

The architect is Mr. Samuel Evans, of Mold, and the contractor is Mr. A. B. Lloyd, of Flint.

## Figures and Tables

**Figure f1:**